# Antioxidant Action of Mangrove Polyphenols against Gastric Damage Induced by Absolute Ethanol and Ischemia-Reperfusion in the Rat

**DOI:** 10.1100/2012/327071

**Published:** 2012-04-30

**Authors:** Felipe Meira de-Faria, Ana Cristina Alves Almeida, Anderson Luiz-Ferreira, Christiane Takayama, Ricardo José Dunder, Marcelo Aparecido da Silva, Marcos José Salvador, Patrícia Verardi Abdelnur, Marcos Nogueira Eberlin, Wagner Vilegas, Walber Toma, Alba Regina Monteiro Souza-Brito

**Affiliations:** ^1^Departamento de Farmacologia, Faculdade de Ciências Médicas, UNICAMP, 13083-970 Campinas, SP, Brazil; ^2^Departamento de Fisiologia e Biofísica, Instituto de Biologia, UNICAMP, 13083-970 Campinas, SP, Brazil; ^3^Departamento de Química Orgânica, Instituto de Química, UNESP, Araraquara, SP, Brazil; ^4^Departamento de Biologia Vegetal, Instituto de Biologia, UNICAMP, 13083-970 Campinas, SP, Brazil; ^5^Departamento de Química Orgânica, Instituto de Química, UNICAMP, 13083-970 Campinas, SP, Brazil; ^6^Faculdade de Farmácia, Universidade Santa Cecília, UNISANTA, Santos, SP, Brazil

## Abstract

*Rhizophora mangle*, the red mangrove, has long been known as a traditional medicine. Its bark has been used as astringent, antiseptic, hemostatic, with antifungic and antiulcerogenic properties. In this paper, we aimed to evaluate the antioxidant properties of a buthanolic fraction of the *R. mangle* bark extract (RM) against experimental gastric ulcer in rats. Unib-Wh rats received pretreatment of *R. mangle* after the induction of gastric injury with absolute ethanol and ischemia-reperfusion. Gastric tissues from both methods were prepared to the enzymatic assays, the levels of sulfhydril compounds (GSH), lipid peroxides (LPO), and the activities of glutathione reductase (GR), glutathione peroxidase (GPx), superoxide dismutase (SOD) and myeloperoxidase (MPO) were measured. The RM protected the gastric mucosa in both methods used, ethanol-induced gastric ulcer and ischemia-reperfusion, probably, by modulating the activities of the enzymes SOD, GPx, and GR and increasing or maintaining the levels of GSH; in adittion, LPO levels were reduced. The results suggest that the RM antioxidant activity leads to tissue protection; thus one of the antiulcer mechanisms present on the pharmacological effects of *R. mangle* is the antioxidant property.

## 1. Introduction

Oxygen-derived free radicals have recently been postulated to play an important role in the pathogenesis of acute gastric mucosal injuries induced by ischemia-reperfusion and ethanol in rats [1]. Ischemia and reperfusion are known to induce gastric lesions, predominantly due to excessive formation of reactive oxygen metabolites and adhesion of neutrophils to endothelial cells. Ischemia weakens the gastric mucosal barrier and increases the acid back-diffusion predisposing the gastric mucosa to damage. After reperfusion, the reactive oxygen species (ROS) are generated from the xanthine-xanthine oxidase system and activated neutrophils, leading to tissue lipid peroxidation (LPO), which in combination with gastric secretion results in damage and cellular death [2]. ROS are involved in the ethanol-induced mucosal damage leading to oxidative stress. In order to protect tissues against the damage provoked by ROS, all cells contain antioxidant enzymes, including glutathione peroxidase (GPx), catalase (CAT), superoxide dismutase (SOD), glutathione reductase (GR), and radical scavengers, such as sulfhydryl compounds (GSH) [3].

Plant polyphenols are known to inhibit LPO and lipoxygenases, and information has been accumulated over the past few years demonstrating their ability to scavenge radicals such as hydroxyl, superoxide, and peroxyl, which are known to be important in cellular prooxidant states [4]. Mangrove plants are well-known as a rich source of tannins [5], which may come from a variety of different *Rhizophora *species [6]. Tannins are known to exist in *R. apiculata* and *R. mangle* but their chemical, biological, and pharmacological properties have not yet been completely determined. The mangrove tannins have substantial reducing power that is comparable to the synthetic standards and other commercial tannins evaluated [7].

In various Caribbean countries, *R. mangle*, the red mangrove, has long been known as a traditional medicine. According to Roig [8], its bark has been used as astringent, antiseptic, hemostatic, with antifungic and antiulcerogenic properties. In recent years, various authors described some activities such as antibacterial [9], antioxidant [10–12], antiulcer [13, 14], and wound healing [15, 16], as well as anti-inflammatory [17].

*R. mangle *is under study in our laboratory, and in a previous work [14], its efficacy was tested in ethanol-induced gastric lesions for three fractions obtained from acetone-water (7 : 3) bark extract. The buthanolic fraction (RM) showed more significant activity at a dose of 0.5 mg kg^−1^. Thus, in this study, we aimed to investigate the phytochemicals of RM, their radical scavenging potential, and its antioxidant properties in ulcer models.

## 2. Material and Methods

### 2.1. Drugs and Chemicals

Lansoprazole (Medley, Brazil); NaCl (Chemco, Brazil); Ethanol, *n*-Buthanol, Ethyl Acetate, Acetone, (Merck, Germany); Ketamine (Fort Dodge, EUA); Xylazine (König, Argentina); Gallic Acid and Quercetin (Sigma-Aldrich, Germany). All drugs were prepared immediately before use.

### 2.2. Animals

Unib-WH male rats (180–250 g), obtained from the breeding of the Universidade Estadual de Campinas (CEMIB/UNICAMP), were used. Animals were fed with a certified Nuvilab CR-diet, with free access to tap water, and were housed on a 12 h light/dark cycle at 60 ± 1% humidity and a temperature of 21 ± 2°C.

### 2.3. Plant Material

The barks of *Rhizophora mangle* L. were collected in “Estuário de Santos”, Santos/SP, Brazil. Professor Msc. Paulo Salles Penteado Sampaio authenticated the botanical identity of the plants and a voucher specimen (HUSC-P.S.P. Sampaio et al., 800) was deposited to the “Herbário da Universidade Santa Cecília-HUSC.”

### 2.4. Preparation of Extracts and Fractions

The bark of *R. mangle* was dried for seven days at 40°C (213 g) and powdered (3 *μ*m). The acetone : water (7 : 3) extract was prepared by maceration and obtained a yield of 31.4% (66.9 g). The extract from *R. mangle* bark (CE) was submitted to liquid-liquid partition with solvents of increasing polarity, thereby semipurified fractions. This methodology provides a proper clean up of the polar extracts [18]. Approximately 20 g of the extract was partitioned between 150 mL of water and 50 mL of ethyl acetate in a separation funnel for 3 times. Then, the aqueous phase was also partitioned with 50 mL of *n*-butanol (for 3 times). All fractions were concentrated under vacuum to obtain the dried fractions: aqueous fraction (Aq; 6.34 g, 31.7%), ethyl acetate fraction (EtOAc; 4.82 g, 24.10%), and butanolic fraction (BuOH; 7.73 g, 38.65%).

### 2.5. Determination of Total Phenols (Folin-Ciocalteu)

The concentration of total phenols RM was determined with Folin-Ciocalteu reagent following the colorimetric method adapted by Huang and co-workers [45]. Measurements were carried out in triplicate, and calculations were based on a calibration curve obtained with gallic acid. The levels of total phenols were expressed as milligrams of gallic acid equivalents per gram of weight (mg GAE g^−1^ W).

### 2.6. DPPH Radical Scavenging and Evaluation of Antioxidant Capacity by ORAC Assay

Scavenging of the stable DPPH radical (2,2-diphenyl-1-picrylhydrazyl) was assayed *in vitro* [19, 20] and the absorbance was measured at 517 nm. Percentage inhibition was calculated from the control. Quercetin was used as a positive control. The antioxidant capacity of Butanolic fraction (RM) from *R. mangle* was assessed through the oxygen radical absorbance capacity (ORAC) assay. The ORAC assay measures antioxidant scavenging activity against peroxyl radical using fluorescein as the fluorescent probe. ORAC assays were carried out on a Synergy HT multidetection microplate reader system. The temperature of the incubator was set at 37°C. The procedure was carried out according to the method established by Ou and coworkers [21]. The data were expressed as micromol of Trolox equivalents (TE) per gram of extract or fraction on dry basis (*μ*mol of TE g^−1^) and as relative Trolox equivalent for quercetin used as a positive control. The analyses were performed in triplicate.

### 2.7. Electrospray Ionization-Mass Spectrometry Fingerprint (ESI-MS)

Butanolic fraction (RM) was diluted in a solution containing 50% (v/v) chromatographic grade methanol (Tedia, Fairfield, OH, USA) and 50% (v/v) deionized water and 0.5% of ammonium hydroxide (Merck, Darmstadt, Germany). ESI-MS fingerprints in the negative ion mode of RM were acquired and accumulated over 60 s, and spectra were scanned in a range between *m*/*z* 100 and 1000, using a Micromass-Waters Q-TOF mass spectrometer (Waters, Manchester, England). Capillary and cone voltages were set at −3000 V and −40 V, respectively, with a desolvation temperature of 100°C. ESI-MS was performed by direct infusion with typical flow rate of 10 *μ*L min^−1^ using a syringe pump (Harvard Apparatus, MA, USA). The RM constituents were identified by comparison of their MS/MS data with data from the literature [5–7, 22].

### 2.8. Ethanol-Induced Gastric Lesions

Ethanol-induced ulcers were evaluated in rats according to Morimoto et al. [23]. Rats fasted for 24 h were treated orally with RM (0.5, 1.5 and 3.0 mg kg^−1^) or lansoprazole (30 mg kg^−1^) or 0.9% saline (10 mL kg^−1^) (RM and lansoprazole were diluted in 0.9% saline solution—used as vehicle). One hour after treatment, all the animals received 1 mL of absolute ethanol, given orally. Animals were killed 1 h after ethanol administration and their stomachs removed and analyzed as subscribed.

### 2.9. Ischemia-Reperfusion-Induced Gastric Lesions

Ischemia-reperfusion damage was produced in rats by a method proposed by Ueda et al. [24]. Rats fasted for 24 h were treated orally with RM (0.5 mg kg^−1^) or lansoprazole (30 mg kg^−1^) or 0.9% saline (10 mL kg^−1^). After one hour, the rats were anaesthetized by intramuscular injection of Ketamine (50 mg kg^−1^)/Xylazine (10 mg kg^−1^). The left side of the abdomen was shaved, and an incision was made. Briefly, the celiac artery was dissected, free of fat excess, and clamped for 30 min (ischemia phase) using a micro-bulldog clamp. Reoxygenation was allowed by removal of the clamp for 60 min (reperfusion phase).

### 2.10. Data Capture and Preparation of Samples for Biochemical Assays

At the end of each experiment, animals were killed by cervical dislocation, the stomachs removed, opened along the great curvature, and fixed between two glass plates. The inner surface of the stomach was photographed with a Nikon Coolpix 4500 camera for later computer analysis. The total ulcerated area in the stomach corpus was measured with Bioview 4 AvSoft, Brazil [25]. Subsequently, the mucosa of each stomach was scrapped off using two glass slices with ice, homogenized in phosphate buffer (0.1 M, pH 7.4), and frozen at −80°C until biochemical determinations. The protein concentration of the samples was determined following the method described by Bradford [26].

### 2.11. Levels of Sulfhydryl Contents (GSH)

GSH levels of gastric tissue of animals were determined by Ellman's reaction using 5′5′-dithio-bis-2-nitrobenzoic acid (DTNB) as described by Faure and Lafond [27]. The intensity of the yellow colour was read at 412 nm.

### 2.12. Glutathione Peroxidase Activity (GPx)

GPx activity was quantified by following the decrease in absorbance at 365 nm induced by 0.25 mM H_2_O_2_ in the presence of reduced glutathione (10 mM), NADPH, (4 mM), and 1 U enzymatic activity of GR [28].

### 2.13. Glutathione Reductase Activity (GR)

GR activity was measured according to Carlberg and Mannervick [29], following the decrease in absorbance at 340 nm induced by oxizied glutathione in the presence of NADPH in phosphate buffer, pH 7.8. Absorbance changes were read between 1 and 10 min.

### 2.14. Superoxide Dismutase Activity (SOD)

SOD activity was analyzed by the reduction of nitroblue tetrazolium using a xanthine-xanthine oxidase system, that is, superoxide generation [30].

### 2.15. Myeloperoxidase Activity (MPO)

MPO activity in the gastric mucosa was measured by the method proposed by Krawisz et al. [31], with minor modifications in Farias-Silva et al. [3], to evaluate neutrophil accumulation. Briefly, the samples were centrifuged at 3000 ×g for 15 min at 4°C. Aliquots of the supernatant were then mixed with a reaction buffer of 50 mM phosphate buffer, pH 6.8, containing 0.005% H_2_O_2_ and 1.25 mg mL^−1^ *o*-dianisidine dihydrochloride, measured at 460 nm.

### 2.16. Estimation of Lipid Peroxidation (LPO)

The homogenate of the glandular portion of stomach was diluted in 0.15 M KCl (ratio 1 : 10). Then to 0.5 mL of this homogenate were added 0.2 mL of dodecyl sulfate (8.1%), 1.5 mL of acetic acid (20%, adjusted with NaOH solution to pH 3.5), 1.5 mL thiobarbituric acid (0.8% w/v), and 0.3 mL of distilled water. All samples were left in water bath with thermostat set at 95°C for 1 hour. After this period, the samples were cooled and added to 1 mL of distilled water and 5 mL of the mixture *n*-buthanol + pyridine (15 : 1, v/v), shaken in vortex for 1 min, and centrifuged at 1400 G for 10 minutes. The absorbance of organic layer was determined, to 532 nm. TEPP (1,1,3,3-tetraethoxypropane) diluted in ethanol was used as standard. The results were expressed as picomoles of substances that react with thiobarbituric acid (TBARS) per mg of protein (nmol TBARS mg protein^−1^) [32].

### 2.17. Statistical Analysis

Results were expressed as the mean ± s.d. or s.e.m., and statistical significance was determined by one-way analysis of variance (ANOVA) followed by Dunnett's *post hoc* test, with the minimum level of significance set at *P* < 0.05*.

## 3. Results

### 3.1. Electrospray Ionization-Mass Spectrometry Fingerprint (ESI-MS)

**Figure 1:**
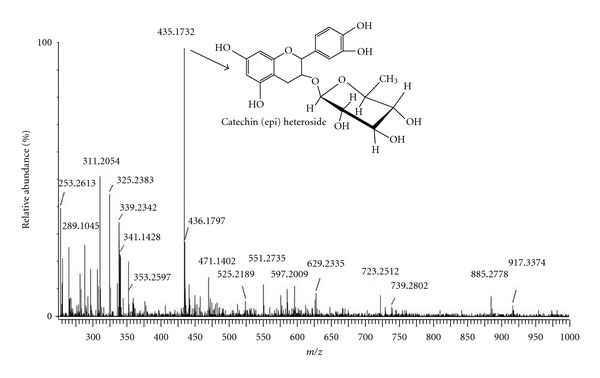
ESI/MS negative mode mass spectra fingerprint of the tannins of *R. mangle* concentrated in the buthanolic fraction (RM). The molecule presented is a condensed tannin monomer which is the relative major molecule in RM identified as catechin (epi) heteroside.

The ESI-MS analysis allowed the profile of molecules found in RM. It was verified that the relative major constituent of the RM is a condensed tannin monomer identified as catechin (epi) heteroside (Figure 1).

### 3.2. Effects of RM on Ethanol-Induced Gastric Lesions

**Figure 2:**
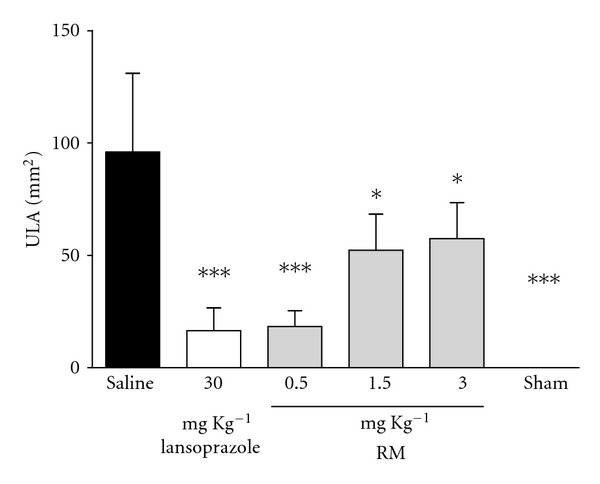
Effect of pretreatment with RM on ethanol-induced gastric ulcer in rats. The animals received 0.9% saline solution (vehicle), lansoprazole (30 mg kg^−1^), and RM (0.5, 1.5, and 3.0 mg kg^−1^); *sham* group represents the manipulated animals. The results are expressed as mean ± s.d. (*n* = 8), and statistical significance was determined by one-way analysis of variance (ANOVA) followed by Dunnett's *post-hoc t* test, (*P* < 0.05* and *P* < 0.001***).

The oral pretreatment with RM (0.5, 1.5, and 3.0 mg kg^−1^) significantly reduced the ulcerative lesion area (ULA) provoked by the administration of absolute ethanol (10 mL kg^−1^) by 80.93, 45.53, and 40.18%, respectively, while lasoprazole presented 82.88% (Figure 2). The data obtained revealed that the RM at 0.5 mg kg^−1^ showed the same effect of the lansoprazole at a standard dose of 30.0 mg kg^−1^, this represents a sixtyfold lower dose than usually used. Thus the dose of 0.5 mg was chosen for further study of the antioxidant activity.

### 3.3. Effects of RM on Ischemia-Reperfusion-Induced Gastric Lesions

**Figure 3:**
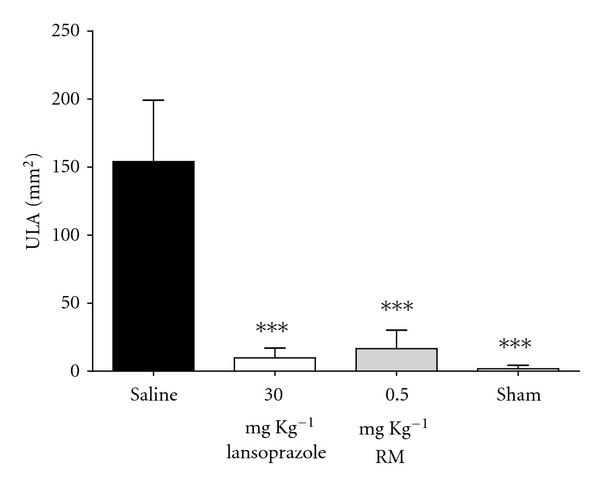
Effect of pretreatment with RM on ischemia-reperfusion-induced gastric ulcer in rats. The animals received 0.9% saline solution (vehicle), lansoprazole (30 mg kg^−1^), and RM (0.5 mg kg^−1^); *sham* group represents the manipulated animals. The results are expressed as mean ± s.d. (*n* = 8), and statistical significance was determined by one-way analysis of variance (ANOVA) followed by Dunnett's *post hoc t* test, with the level of significance set at *P* < 0.001***.

In the ischemia-reperfusion model of gastric injury (Figure 3), again there was similar gastroprotective activity between lansoprazole (93%) and RM (89%); however, it is worth emphasizing the difference between the doses, although it was a surprise to find such efficacy of lansoprazole in this model.

### 3.4. Antioxidant Assay

#### 3.4.1. Radical Scavenging (DPPH), ORAC-FL Assays, and Phenolic Compounds Contents (Folin Ciocalteu)

RM showed EC_50_ radical scavenging activity at 8.065 *μ*g mL^−1^, while the positive control quercetin that was used as standard obtained same values at 2.924 *μ*g mL^−1^. ORAC results for analyzed samples and results for quercetin (reference compound) are summarized in Table 1. This finding is consistent and may be related to the concentration of phenolic compounds found in RM through Folin-Ciocalteu reaction (226.7 mg of GAeq g^−1^).

#### 3.4.2. Effects of RM on GSH and LPO Levels

The administration of ethanol provoked a decrease in GSH levels (59%) and enhanced the LPO (30%); ischemia-reperfusion reduced the levels of GSH in 42% while LPO increased by 37%. The pretreatment of RM prevented the decrease observed in GSH levels observed in the ischemia-reperfusion, in addition increased GSH in the ethanol model, while LPO levels were maintained at the reference levels (sham group) in both models.

#### 3.4.3. Effects of RM on GR, GPx, and SOD Activities

The administration of ethanol decreased the activity of GR (46%), GPx (51%), and SOD (92%). No differences between saline and sham group were found in the activity of these enzymes by ischemia-reperfusion method, although the pretreatment with RM significantly increased these activities. The results indicated that RM was able to maintain the baseline enzyme activity levels in the ethanol-induced gastric ulcer while increasing these in the ischemia-reperfusion; also lansoprazole augmented the activity of SOD (87%) in the ischemia-reperfusion model.

#### 3.4.4. Effect of RM on MPO Activity

The activity of MPO was found elevated by 94% in the ischemia-reperfusion and by 59% in the ethanol model. The oral administration of RM maintained the MPO activity at the baseline in both methods, and lansoprazole was significant in the ethanol model.

## 4. Discussion

The most common classes of condensed tannins are the procyanidins, which are chains of catechin, epicatechin, and their gallic acid esters, and the prodelphinidins, which consist of gallocatechin, epigallocatechin, and their galloylated derivatives as the monomeric units. In mangrove species, condensed tannins are abundant components (as high as 20% dry weight), which prevent damage from herbivores, but they also show a diversity of other biological activities of historical and potential importance to humans [22].

Tannins are potent scavengers of peroxyl radicals and can also interact with mucus proteins, improving their cytoprotective effect by forming a protein lining over the gastrointestinal mucosa [33]. The mangrove tannins have substantial reducing power, DPPH as well as ABTS-free radical-scavenging abilities, that are comparable to the synthetic standards and other commercial tannins evaluated [7].

Folin-ciocalteu, DPPH, and ORAC assay reveals high concentration of tannins in the RM, which demonstrated potent radical scavenging activity (Table 1). The fingerprint of RM showed the presence of condensed tannin monomers as major compounds, such as (epi) catechin and catechin (epi) heteroside (Figure 1). These molecules possess highly antioxidant properties as demonstrated here. It was reported that the glycoside moiety in condensed tannins structure can enhance the effectiveness of condensed tannins radical scavenging [34]. Zhang et al. [22] showed the DPPH free radical scavenging activity of the mangrove condensed tannins and the references at different concentrations, and demonstrated that all tested antioxidants showed dose-dependent activity. The free radical scavenging activity increased with the increasing concentration of condensed tannins.

In this work, we found an interesting antioxidant activity, as mentioned above, which we supposed to be responsible for the antiulcer effects. Thus, we studied two models of induced ulcer, ethanol (Figure 2) and ischemia-reperfusion (Figure 3), in which prooxidant molecules play an important role in the pathophysiology. The administration of RM (0.5; 1.5 and 3.0 mg·kg^−1^) promoted gastroprotection against the effects of absolute ethanol on the gastric mucosa. An interesting finding can be observed in Figure 2; the dose of 0.5 mg·kg^−1^ of RM was more efficient than 1.5 and 3.0 mg·kg^−1^, this could be explained by the presence of glycosylated compounds, which improve the absorption of these compounds, suggesting that the substances presented in the RM need lower concentrations to promote its pharmacological activities. Thus the subsequent model (ischemia-reperfusion) was done using the dose of 0.5 mg·kg^−1^ of RM.

Gastric lesions by ethanol may be associated with ROS generation; these lesions produce an imbalance between oxidant and antioxidant cellular processes [35, 36]. Currently, there is consensus that the deleterious effects of ethanol on gastric mucosa are consequence of the enhanced lipid peroxidation and decreased glutathione levels [37]. On the other hand, in ischemia-reperfusion model, the injuries occur without the use of chemical agents [38]. Ischemia weakens the gastric mucosal barrier by increasing the diffusion of acid, which causes damage to the mucosa [39]. During ischemia, there is a reduction of blood flow in the body leading to a sequence of chemical reactions that result in dysfunction, cell necrosis, and the appearance of toxic metabolites contributing to cell death [40]. With reperfusion, ROS are generated, especially in the xanthine oxidase system and neutrophil activation, causing lipid peroxidation in the tissue, hence, the combination of ROS with acid secretion promotes damage in the gastric mucosa [41]. Gastric lesions occurring in the reperfusion phase are considered more severe than those occurring during the ischemia, since there is participation of ROS, including biomolecules as O_2_^●^, OH^●^, and H_2_O_2_, which attack membrane lipids, nucleic acids, enzymes, and receptors, causing changes in structure, cellular activity, and transport of proteins. To be protected against oxidative injury, cells evolved complex cellular defense mechanisms and the capability to use exogenous antioxidants to eliminate ROS. The potential role of micronutrients as antioxidants (vitamin C, vitamin E, carotenoids, and polyphenols) has stimulated intense research efforts [42].

The imbalance between ROS and antioxidant defense leads to oxidative modification in cell membrane or in intracellular molecules [43]. The first gastric mucosa antioxidant enzyme is SOD, which catalyses the dismutation of O_2_^●^ into H_2_O_2_, which is less harmful; the second step in the H_2_O_2_ metabolism depends on the activity of GPx. The reduction of H_2_O_2 _to water by GPx is accompanied by the conversion of glutathione in the reduced form (GSH) to oxidized form (GSSH), which is then converted to GSH by the GR [44].

The synthesis of glutathione is determined enzymatically by *γ*-glutamylcysteine synthase (*γ*-GCS) and glutathione synthase; this formation is limited by these enzymes activities. An important task of cellular glutathione is the free radical and peroxides sequestration generated during the cell respiration process that can lead to oxidation of proteins, lipids and nucleic acids. Compensatory mechanism of oxidative damage involves the transactivation of genes responsible for enzymes involved in the synthesis and metabolism of glutathione [45]. Moskaug et al. [46] showed that the administration of polyphenols such as quercetin induces *γ*-GCS expression and consequently increases intracellular glutathione. Thus, it was observed that the compounds present in the RM induce the synthesis of glutathione, acting by increasing (Table 2) as well as maintaining (Table 3) the levels of GSH, allegedly by this mechanism.

As mentioned above, GPx is an enzyme that plays a fundamental role in the elimination of hydrogen peroxide and lipid hydroperoxides in the gastric mucosa cells [37]; the antioxidant activity of GPx is coupled with the oxidation of reduced glutathione (GSH), which subsequently can be reduced by GR using NADPH as reducing agent. Increased levels of SOD in response to noxious stimuli play an important role in the protection of oxidative stress [47]. Berenguer et al. [11] reported that the crude extract of *R. mangle *augmented the activity of SOD and GPx in a model of gastric ulcer induced by diclofenac. In addition, the author has found a decrease in lipid peroxidation *in vitro*, these findings are in accordance to our data; however, there is a strong difference between doses used in both studies. In the present study, the dose used was 125-fold lower than the active dose in that study. Moreover, this information may be explained by the fractionation of the extract, which leads to the concentration of compounds capable of promoting the antioxidant effect at lower doses in the buthanolic fraction (RM).

The lipid peroxidation mediated by ROS is an important cause of destruction and damage to cell membranes, and it is involved in the pathogenesis of acute mucosal injury induced by ethanol and ischemia-reperfusion [37]. RM was able to reduce the levels of LPO obtained in the models studied (Tables 2 and 3); this reduction is probably by the elimination of these compounds by GPx; once its activity was found to be increased.

The MPO is described as a marker of the infiltration/aggregation of neutrophils and is often increased in ulcerogenic lesions [48]. Neutrophils represent the first line of innate immune defense to phagocytosis, killing and digesting bacteria and fungi; the enzyme NADPH oxidase is an essential component of neutrophils, which is responsible for the free radical generation and other ROS such as superoxide anion [49]. Neutrophils are the major mediators to increase the microvascular permeability induced by reperfusion [50]; some authors have reported that the exposure of gastric mucosa to ethanol and ischemia followed by reperfusion caused significant increase in the MPO activity [48]. Thus, the MPO activity was studied in both models as oxidant component of the gastric mucosa (Tables 2 and 3). The data obtained indicated possible antioxidant mechanism promoted by the compounds of the RM, since reduction was observed in MPO activity, showing the antioxidant activity presented by RM.

## 5. Conclusion

The results obtained in ethanol and ischemia-reperfusion models argue that the rise and maintenance of GSH may have generated conditions to SOD, GPx, and GR present their regular activities. Therefore, the compounds present in the RM demonstrated that its antioxidant action could be achieved by modulating glutathione and the subsequent activity of SOD, GPx, and GR, reducing LPO levels and MPO activity, promoting gastroprotection.

## Figures and Tables

**Table 1:** RM radical scavenging on DPPH, antioxidant capacity in ORAC-FL assay, and fractions and phenolic compounds content in Folin-Ciocalteu.

Treatments	DPPH EC_50_^a^ (*μ*g mL^−1^)	ORAC assay^a, b^ (*μ*mol of TE g^−1^)	Folin-Ciocalteu^a, c^ (mg of GAE g^−1^)
Quercetin	2.924 ± 1.666	5.62 ± 0.690^d^	—
RM	8.065 ± 2.369	8611.19 ± 0.312	226.7 ± 2.655

^a^Mean ± standard deviation of triplicate assays.

^b^ORAC data expressed as micromol of Trolox equivalents per gram (*μ*mol of TE/g).

^c^Total phenolics data expressed as milligrams of gallic acid equivalents per gram (mg of GAE/g).

^d^Positive control, ORAC data expressed as relative Trolox equivalent, mean ± standard deviation of triplicate assays.

**Table 2:** Effect of RM on the antioxidant compounds and enzymes in ethanol-induced gastric ulcer in rats.

Treatments	Dose (mg kg^−1^)	GSH (nmol mg of protein^−1^)	SOD (U mg of protein^−1^)
Saline	10 (mL kg^−1^)	5.88 ± 0.379	2.24 ± 1.315
Sham	—	14.58 ± 1.091*	28.82 ± 2.888***
Lansoprazole	30	10.53 ± 0.867	4.29 ± 3.006
RM	0.5	21.44 ± 0.956***	21.87 ± 3.314***

Treatments	Dose (mg kg^−1^)	GPx (nmol min^−1^ mg of protein^−1^)	GR (nmol min^−1^ mg of protein^−1^)

Saline	10 (mL kg^−1^)	50.01 ± 4.248	11.00 ± 1.220
Sham	—	103.8 ± 5.556***	20.56 ± 2.995***
Lansoprazole	30	61.17 ± 4.976	11.26 ± 1.067
RM	0.5	96.66 ± 3.550***	16.94 ± 1.305*

Treatments	Dose (mg kg^−1^)	LPO (nmol TBARS mg of protein^−1^)	MPO (U mg of protein^−1^)

Saline	10 (mL kg^−1^)	2.98 ± 0.007	2.51 ± 1.013
Sham	—	2.29 ± 0.0280*	1.57 ± 1.730**
Lansoprazole	30	2.47 ± 0.307	1.74 ± 0.233*
RM	0.5	2.14 ± 0.117**	1.75 ± 0.136*

The results are expressed as mean ± s.e.m. (*n* = 8); statistical significance was determined by one-way analysis of variance (ANOVA) followed by Dunnett's *post hoc t* test (*P* < 0.05*, *P* < 0.01** and *P* < 0.001***).

**Table 3:** Effect of RM on the antioxidant compounds and enzymes in ischemia-reperfusion-induced gastric ulcer in rats.

Treatments	Dose (mg kg^−1^)	GSH (nmol mg of protein^−1^)	SOD (U mg of protein^−1^)
Saline	10 (mL kg^−1^)	13.39 ± 1.923	10.61 ± 0.304
Sham	—	23.26 ± 0.802**	13.42 ± 1.987
Lansoprazole	30	18. 91 ± 2.036	25.14 ± 2.363***
RM	0.5	20.93 ± 1.535*	18.48 ± 1.882*

Treatments	Dose (mg kg^−1^)	GPx (nmol min^−1 ^mg of protein)	GR (nmol min^−1^ mg of protein^−1^)

Saline	10 (mL kg^−1^)	13.58 ± 1.011	18.00 ± 1.015
Sham	—	15.60 ± 1.612	18.44 ± 0.888
Lansoprazole	30	13.47 ± 0.967	18.94 ± 0.705
RM	0.5	21.96 ± 1.917**	26.86 ± 2.341**

Treatments	Dose (mg kg^−1^)	LPO (nmol TBARS^−1^ mg of protein^−1^)	MPO (U mg of protein^−1^)

Saline	10 (mL kg^−1^)	2.43 ± 0.111	2.43 ± 0.127
Sham	—	1.77 ± 0.117*	1.25 ± 0.168***
Lansoprazole	30	2.23 ± 0.176	2.21 ± 0.079
RM	0.5	1.758 ± 0.141*	1.86 ± 0.128*

The results are expressed as mean ± s.e.m. (*n* = 8), statistical significance was determined by one-way analysis of variance (ANOVA) followed by Dunnett's *post hoc t* test, (*P* < 0.05*, *P* < 0.01**, and *P* < 0.001***).
